# Novel Insight into How Nurses Working at PH Specialist Clinics in Sweden Perceive Their Work

**DOI:** 10.3390/healthcare8020180

**Published:** 2020-06-19

**Authors:** Bodil Ivarsson, Barbro Kjellström

**Affiliations:** 1 Office of Medical Services, University Trust, Region Skåne, SE-221 85 Lund, Sweden; 2 Department of Cardiothoracic Surgery, Clinical Sciences, Lund University, SE-221 85 Lund, Sweden; 3 Department of Medicine, Karolinska Institute, SE-171 76 Stockholm, Sweden; barbro.kjellstrom@ki.se; 4 Department of Clinical Physiology, Clinical Sciences, Lund University, SE-221 85 Lund, Sweden; 5 Department of Clinical Physiology, Skåne University Hospital, SE-221 85 Lund, Sweden

**Keywords:** advocacy, allied health occupations, health information management, clinical decision-making, communication, chronic disease, holistic care, professionals-patient relations, significant others, team care

## Abstract

Outpatient pulmonary hypertension (PH) specialist centers have an important role in the optimal management of pulmonary arterial hypertension (PAH) and chronic thromboembolic pulmonary hypertension (CTEPH). The aim of the present study was to gain an understanding of the work facing nurses at the outpatient PH specialist centers in Sweden. All nurses (*n* = 14) working at the outpatient PH specialist centers in Sweden were included. Qualitative content analysis was employed to analyze the interviews, wherein an overarching theme emerged: “Build and maintain a relationship with the patient”. Three categories described the nurses’ experiences: “Ambiguous satisfaction regarding information and communication”, “Acting as a coordinator” and “Professional and personal development”. To provide good patient care, the nurses described the key components as the ability to give information on all aspects of the disease and their availability by phone for patients, their relatives, and other healthcare resources. This requires evidence-based, specialist knowledge about the disease, its care, and treatments as well as experience. In conclusion, working as a nurse at the outpatient PH specialist centers highlight the advantages, expectations, and difficulties in working with patients with a rare and life-threatening illness. The overall knowledge and skills were high, but the nurses expressed a need for in-depth and continued training.

## 1. Introduction

Pulmonary arterial hypertension (PAH) and chronic thromboembolic pulmonary hypertension (CTEPH) are rare forms of pulmonary hypertension (PH). In Sweden 2019, approximately 500 patients lived with PAH and 250 with CTEPH, representing 50 and 25 patients per million inhabitants, respectively [1]. Patients are being diagnosed with PAH at a higher age than previously, but still, a third of the patients are diagnosed at an age of 50 or younger [1]. Symptoms such as dyspnea and fatigue are common and often affect the patients’ daily life [2,3,4]. Advances in drug therapy have improved survival and quality of life, but there is still much to be gained before a cure is available [5]. The diseases are complex and there is a high need for individualized care, and this need will continue as the disease progresses [6,7].

Patients with PAH and CTEPH are cared for by a multidisciplinary team at PH specialist centers [8,9]. The diseases have a significant impact on the psychological, social, economic, emotional, and spiritual function of both patients and their next of kin [3,7,10]. For nurses working at the PH specialist centers, their role includes gathering and giving information, coordination of care, assessment, and intervention, education, facilitating research, and last but not least, patient advocacy [9,11,12]. The nurses must work holistically and involve the patient’s family and friends when possible [10]. In addition to the PH specialist centers, the patients will need contact with the primary care [13], the emergency department [14], and the municipality and social insurance services [4]. The PH nurse will be the focal point in this web of healthcare providers and authorities [11].

The present study aimed to gain an understanding of the work facing nurses at the outpatient PH specialist centers in Sweden.

## 2. Material and Methods

### 2.1. Design

This study used a qualitative, descriptive, and retrospective design utilizing conventional content analysis on the collected data [15]. The study was approved by the Regional Ethical Board in Lund, Sweden (Dnr LU 2011/364). The informants were informed that their responses would be kept confidential and that they could withdraw from the study at any time with no explanation. Written informed consent was obtained from all informants before the interviews. The collected data were securely stored on access-restricted devices accessible to the research team only.

### 2.2. Study Setting and Population

All nurses (12 females and 2 males) working at the outpatient PH specialist centers in Sweden were included in the study. A contact list (e-mail) was obtained from the Swedish Association for Pulmonary Hypertension where staff from all the PH clinics in Sweden are members. The mean age of the participants was 53 ± 9 (min 35, max 65) years. On average they had 25 ± 9 years (min 11, max 42) of experience working as a nurse and of those years, 11 ± 6 (min 2, max 29) with PAH and CTEPH. The individual interviews were conducted either as direct face-to-face interviews (*n* = 3) or as telephone interviews (*n* = 11), between December 2018 and April 2019. The length of the interviews varied between 19 to 60 min.

### 2.3. Data Collection 

The nurses were informed by e-mail about the study and then contacted by phone and asked if they agreed to participate. The interviews were semi-structured and covered demographic details such as age, education, and years of experience in PH care. This was followed by an opening question “In your work as a nurse at the outpatient PH specialist centers, what do you do to achieve good care for the patients and what do you think can be done better?” Probing questions were asked to follow-up on the nurses’ responses, clarification of answers and to continue the conversation. One test interview was conducted to validate the opening question and the procedure. No changes were needed after the test interview. All interviews were undertaken in a dialogue form, digitally recorded and then transcribed.

### 2.4. Data Analysis

**Figure 1:**
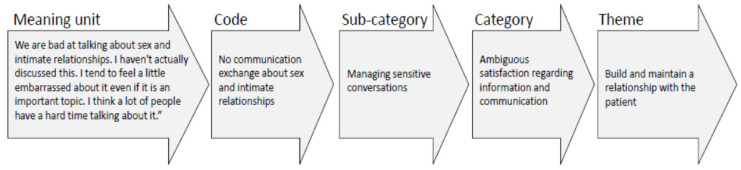
Example of the analysis process, from meaning units to theme.

Transcripts from interviews resulted in 125 double-spaced pages of text and managed using Microsoft Word’s Tools [16]. The transcribed interviews were analyzed through conventional content analysis according to the procedure proposed by Hsieh and Shannon [15]. The transcriptions were read several times to obtain a sense of the whole and divided into meaning units. The number of meaning units provided by each nurse varied between 13 and 29. The units were condensed, coded, sorted into groups with similar content, and abstracted in sub-categories and the first author (BI) did an initial data analysis. Within each sub-category, the statements were critically questioned and discussed, read, and compared to enable an interpretation for identifying and naming categories by both authors (BI and BK). Thereafter, an overall theme was formulated. Both authors discussed the sub-categories and categories and made adjustments until consensus was reached to ensure that the meanings in the nurses’ answers were fully captured. An example of the content analysis process is illustrated in Figure 1. Quotes from the interviews, accompanied by a code in brackets indicating which informant gave the quote, are stated below each category to reinforce the results.

## 3. Findings

**Figure 2:**
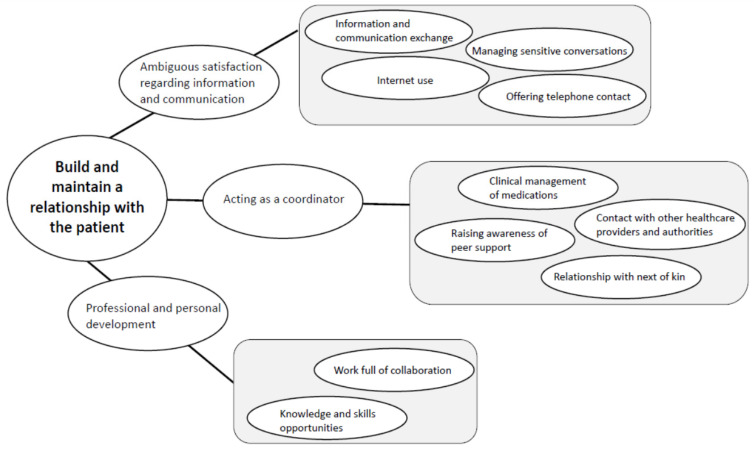
Summary of the subcategories, categories and the main theme.

The overall theme “Build and maintain a relationship with the patient” was considered to capture the nurses’ experiences. Three categories and 10 subcategories were described (Figure 2).

### 3.1. Ambiguous Satisfaction Regarding Information and Communication

#### 3.1.1. Information and Communication Exchange

In the nurses’ experience, there was often a delay before patients were correctly diagnosed and the relief the patients felt when diagnosed often made it difficult for them to absorb more information, regardless if it was oral or written. The information material distributed to patients was largely produced by the pharmaceutical industry, which the nurses tend to perceive as indirect advertising, but it is the only material they had to offer. There is a widespread desire to be able to use neutral information material. Some PH specialist centers had created this kind of material, which the patient can supplement with their own information about the diagnosis, medical test, and medicines and that can be used when the patient meets other healthcare providers. Some suggested a mobile application as an option for sharing information. Overall, the nurses emphasized the importance of individualizing information and repeating it regularly. Additionally, it was important to ask the patient to, in their own words, to say what they remembered about the given information.

“For every patient, I find that you have to tailor the information a little and get a sense of what they can take in.”

(12)

#### 3.1.2. Managing Sensitive Conversations

All nurses stated that physicians are the ones responsible for giving information about difficult and sensitive topics. Questions about the heredity of PH were responded with information that it is rare but, when needed, a cardio genetic examination could be performed. Regarding prognosis, nurses described that their tasks were more about being responsive to what the patients understood and to clarify and convey hope to them. Conveying hope had become increasingly easier in PH healthcare due to positive developments with new medicines and treatment strategies, e.g., combination treatments, which translate to both enhanced levels of wellbeing and better survival rates. Nurses outlined that when all treatment alternatives were exhausted, patients could usually be transferred to special palliative care, which is available for various medical conditions. However, they also stated that they did not always know that a patient was dying, as they often only had contact with the patient once a year.

Another sensitive subject was the unsuitability and danger for fertile female patients if they were to become pregnant. Nurses would follow-up on the physicians’ information and go into more detail about methods of birth control. At the same time, some of the nurses knew that women with PH had given birth or undergone abortion. They were aware of, and expressed concern over, that pregnancy and giving birth was discussed as a possibility in PH patient Facebook groups worldwide, including patients in Sweden.

Regarding information about sex and intimate relationships, the nurses lacked information material and admitted they more or less never initiated discussions about this topic. In some cases, male patients had raised questions about whether the medication could affect their sexual potency. One nurse had the experience that the patient’s partner asked about advice regarding having sexual intercourse.

“We are bad at talking about sex and intimate relationships. I haven’t actually discussed this. I tend to feel a little embarrassed about it even if it is an important topic. I think a lot of people have a hard time talking about it.”

(6)

#### 3.1.3. Internet Use

According to the nurses, it was largely a question of age as to whether patients sought out information via the Internet or not. Younger patients certainly read about the disease online, and an increasing number of older people are also starting to use social media and the Internet as a source of information. Some nurses warned patients about reading pages online that are not from a reliable source and to check whether the information might be outdated and no longer relevant. Some nurses said they referred patients to “a Swedish specific web-based forum for PH patients” that was considered to be safe and relevant.

“Patients can misinterpret numbers and figures on death statistics… and some patients have been truly devastated to read this information, so it is really beneficial that they can come to us and get the right information”

(7)

#### 3.1.4. Offering Telephone Contact

The nurses believed that good telephone availability for patients was a major factor in healthcare, as care is increasingly given in an outpatient setting. The nurses often contact the patient by telephone when starting a new treatment and/or a titration of medicine. Patients and relatives often contacted nurses over the phone with all sorts of issues, such as treatment side effects, general infections, weight gain, and also, when they did not know who else to contact with concerns besides their PH disease. Sometimes, the nurses could make an assessment by telephone that the patient needed to visit the outpatient PH specialist centers and could then arrange a time directly.

“I try to make follow-up calls with patients frequently but still, over the phone isn’t always the best way, sometimes you just need to sit down and talk face-to-face...”

(12)

### 3.2. Acting As a Coordinator

#### 3.2.1. Clinical Management of Medications

Medication management was considered to be one of the more complex tasks carried out by the nurses. In collaboration with the patient responsible physician, they were highly involved when patients started a new medication and in the phase of dose escalation. They described that some of the medicines have significant side effects, such as nausea and diarrhea, dizziness, headaches, and muscle pain; especially at the beginning of treatment. Nurses emphasized that trust and being able to listen were of the utmost importance when providing advice and support upon problems, as by doing so, they can ensure that patients do not interrupt the course of their treatment. It is also important to build trust so that patients are more likely to inform us if they are using non-prescription drugs, herbal remedies, or other complementary methods. The nurses had a feeling that there was an issue with under-reporting in these areas.

“We tend to be a little mean and tell the patient that we will be happy if they get side effects...it kind of gives the impression that the medicine is working, to make the patients understand that it is nothing to be afraid of.”

(7)

#### 3.2.2. Contact with Other Healthcare Providers and Authorities

Some contact was made with patients’ home hospitals and health centers, and most often in connection with referrals and medical tests. The nurses felt that patients preferred to go to the outpatient PH specialist centers when they were afflicted with an ailment, regardless of whether or not it was related to the PH disease. The nurses often had to guide the patients on where to seek help in the healthcare system and to find the right level of care. Nurses also felt that most working-age patients wanted to work but that they did not have sufficient strength for a full-time job or that symptom such as fatigue and shortness of breath made heavy physical work difficult to perform. The nurses found that, like many healthcare providers, the social insurance agency and employers had insufficient knowledge of PH and the nurses had to support the patients by providing information or arranging medical certificates with extended doctor’s statements from the treating physicians to the patients.

“It is quite a lot of work required from us and the medical team behind the PH patients in talking to physicians, making clarifications, writing new medical certificates, and explaining everything more clearly once more. So, yes, it’s an unnecessary energy thief for us and unnecessary worry and strain for this patient group.”

(1)

#### 3.2.3. Relationship with Next of Kin

The nurses encouraged patients to bring their next of kin, so the patient and their relatives could get the same picture of the exchanged information. But, at the same time, the nurses emphasized that it is the patients who decide whether the next of kin can attend the visits. The nurses were well aware that for some patients, the relatives are also their caregivers, and in these cases, close contact is particularly important regarding medication intake and living habits. One observation the nurses made was an increase in patients that were alone and without supportive social networks.

“We really try to see the relatives as a resource… it is good to share, but in order for a family member to be able to understand things properly, they need to have knowledge of what this is all about and the goals we have in terms of treatment of the illness.”

(1)

#### 3.2.4. Raising Awareness of Peer Support

Nurses stated that they distributed information material from the Swedish PH patient association and conveyed invitations to events organized by the patient association to the patients. When invited by the patient association, the nurses contributed as a lecturer. Some nurses mediated contact between patients, to share their experiences living with the disease; this could be either a representative from the patient association or on a more private basis.

“When it’s been a while, we usually advise our patients to talk to another patient in their own age… We know a lot about the disease, but we haven’t had it ourselves. A fellow patient can explain that in a good, and better way.”

(2)

### 3.3. Professional and Personal Development

#### 3.3.1. Work Full of Collaborations

There is a Swedish PH national quality registry that all nurses came into contact with. They motivated the patients and got their consent to participate in the registry. Some nurses also entered data into the registry. Several nurses reported that when clinical trials and other research took place at the PH specialist clinic, they participated in collecting data for the study procedures. Their experience was that virtually all patients they contacted had a positive attitude to the registry and on research and development. The nurses found that meetings with the PH team, where individual patient cases in regard to diagnosis, treatment, examinations, and care were discussed, had a significant impact on their professional development. Debriefing, regarding problematic or emotionally difficult cases, which often occur in severe chronic illness, was largely absent at the PH team meetings. However, all the nurses felt that they had someone within the team with whom they could share issues of an emotional nature. Some pointed out that the social worker or psychologist associated with the PH team was available for the patients, and not the staff.

“We have a PH team meeting, where the physicians can raise issues if they are hesitant about how to proceed and discuss treatment options. There are cases that are difficult and where you have back-and-forth discussions. It is very educational, and the physicians appreciate getting feedback... it is simply a very good learning opportunity”

(13)

#### 3.3.2. Knowledge and Skills Opportunities

Nurses highlighted the Swedish Association for Pulmonary Hypertension, which is a multidisciplinary organization that consists of physicians, nurses, physiotherapists, curators, and other occupational categories. The association organizes bi-annual meetings for professional exchange. The nurses received a large part of their continuing vocational training by meeting others who work with PH and taking part in research and development and training provided by the medical industry. Still, the lack of academic training opportunities and specialist degrees for nurses in the PH area were also addressed.

“…the Swedish Association for Pulmonary Hypertension network meeting for the nurses is very important... but I would like more possibilities for education, PH is truly a serious and complex disease.”

(10)

## 4. Discussion

This study provides novel insight into how nurses working at outpatient PH specialist centers in Sweden perceive their work. To provide good patient care, the nurses described the key components as the ability to give information on all aspects of the disease and their availability by phone for the patients, their next of kin, and other healthcare resources. This requires evidence-based, specialist knowledge about the disease, its care, and treatments as well as experience, or if newly employed, mentorship from a more experienced colleague. The need for academic education and training for those who cares for patients with rare diseases have been highlighted earlier [9].

In the Nordic countries, we have previously shown that a majority of the PH specialist centers can be reached by telephone five days a week or more [9]. The present study confirmed that the telephone contact facilitated communication and enabled the possibility to give advice, support, and change of treatment, without the patient having to visit the hospital. This is important as phone support can reduce unnecessary emergency department visits, specialist consultations and inpatient admission rates [17].

The ability to provide relevant information directed towards helping patients to understand and manage their disease was emphasized in the present study. Though, the nurses felt that the available educational materials were largely produced by the insurance or drug companies and that this could be detected in the material. They would have liked an unbiased information material and a possibility to individualize the information. In this matter, e-health provided by the healthcare system was mentioned as a means to improve information and support for patients and their families. 

Information about the diseases via the Internet and social media was a concern for the nurses as they know a lot of outdated and irrelevant information is available there. To manage this, inviting the patient to a dialogue about searching for information online was encouraged as there are great potentials for patients and their next of kin to find helpful information on the Internet if used correctly [18]. Peer support and patient associations are valuable resources in providing educational and emotional provision [7,19]. The present study confirmed that the nurse’s relationship with the Swedish PH patient association was appreciated and considered to provide one more dimension of support to the patients. All nurses in the present study considered it to be the physicians’ responsibility to talk to the patient about the prognosis. To fully appreciate what the patients understand about their disease state, the healthcare professionals should ask the patient questions about this [20]. Likewise, all patients should be given the opportunity to discuss goals, hopes, fears, and thoughts about their serious chronic illness and the end of life [20]. The present study provides support for that effective multi-professional teamwork and communication, linked with enhancing, and “team-based” training can improve the quality of care of patients with PH. A core in the work of nurses is planning, implementing, and evaluating information, communication, and educational efforts in dialogue with patients and their next of kin in order to promote health and prevent illness [21]. Therefore, nurses are well-positioned to lead these conversations about difficult topics and at the same time integrate hope, future, and palliative care. However, one should pay attention to the fact that communication training could be needed, either learned formally or acquired from a role model such as a more experienced colleague. 

The PH nurses acknowledged their key role in titrating medications and monitoring side effects [22]. Adherence to medical treatment is a known problem [23,24] and the trust between the nurses and patients were considered essential for the patients to open up about possible problems in this area. Trust is also important in discussions about sexual activity and function [3,7,10]. The nurses in the present study admitted that talking about this topic was not initiated by them and rarely discussed at all. Information material about sex and intimacy in this patient group might be helpful to initiate a discussion.

Patients may live far away from their PH specialist center [9] and in order to provide the patient with the best possible care and support, shared responsibility with other care providers must be established. The PH nurses have a large role in bridging the gap between specialist and primary care [11]. The present study emphasizes this contact and in addition, it highlights the PH nurse role as coordinators, both within and outside the PH team, when patients need support in their interactions with social insurance agencies and health insurance systems. This is important support since it was previously shown that financial burdens caused stress in patients with PAH [25]. To help patients realize that they are not alone, the nurses mediated contact with peers or the PH patient organization, whose responsibility is to support and provide contact with other patients for shared experiences [7].

The work in multidisciplinary teams and the opportunity to attend the biannual meetings organized by the Swedish PH association made the nurses feel their knowledge was up to date with recent developments in PH care. Despite this, there is a need for specialized education and training for nurses working in the area of PH, a problem likely found also for other rare diseases. Large organizations such as European Society of Cardiology or European Respiratory Society may play a pivotal role in providing certified education in the area of PH, similar to their educational programs in other diseases [9]. In order to provide patients with excellent and safe care, it is of utmost importance that the experience and high competence of the PH nurses are retained and continuously developed [6].

### Methodological Considerations

This study was based on interviews with 14 nurses. Though a small number, they included all nurses working at the outpatient PH specialist centers in Sweden. While the study mainly consisted of women this accurately reflect the situation in Sweden where in 2017, only 12% of those working as a nurse were male [26]. All participants had a long experience working as a nurse, however, the time working at the outpatient PH specialist center was more varied. However, it is possible that if some nurse PH specialists had been younger and with less experience this might have affected the findings. Thus, the generalizability of this study might be limited as the study only represents the outpatient PH specialist centers in one country. Furthermore, the open-ended nature of the interviews can be seen as a limitation. While it encourages discussion of themes of interest for the person being interviewed, some experiences of general interest might have been missed. Despite this, the study appears to give a reasonably comprehensive picture of the nurses’ work in the outpatient PH specialist centers. We believe that it could serve to inspire nurses at PH specialist centers or other healthcare professionals, patient organizations and the society to improve the supportive care for both patients with PH and their families.

## 5. Conclusions 

This study elucidates the essential parts of nurses’ views and experiences when working in the outpatient PH specialist centers. It highlights the advantages, expectations, and difficulties in working with patients with PH, a rare and life-threatening illness. The overall knowledge, skills, and understanding of PH care were high among the participating nurses. Nevertheless, nurses expressed a need for in-depth and continued training in understanding the PH disease, treatment, and other care. The result of this study might be useful in professional development and the syllabus for PH nurses.
